# 7T MRI detects widespread brain iron deposition in neuroferritinopathy

**DOI:** 10.1002/acn3.52053

**Published:** 2024-04-01

**Authors:** Alexander G. Murley, Catarina Rua, Heather Biggs, Christopher T. Rodgers, Tomasz Matys, Jelle van den Ameele, Rita Horvath, Patrick F. Chinnery

**Affiliations:** ^1^ Department of Clinical Neurosciences University of Cambridge Cambridge UK; ^2^ Wolfson Brain Imaging Centre University of Cambridge Cambridge UK; ^3^ Invicro London UK; ^4^ Department of Radiology University of Cambridge Cambridge UK; ^5^ MRC Mitochondrial Biology Unit University of Cambridge Cambridge UK

## Abstract

Neuroferritinopathy is a disorder of neurodegeneration with brain iron accumulation that has no proven disease‐modifying treatments. Clinical trials require biomarkers of iron deposition. We examined brain iron accumulation in one presymptomatic *FTL* mutation carrier, two individuals with neuroferritinopathy and one healthy control using ultra‐high‐field 7T MRI. There was increased magnetic susceptibility, suggestive of iron deposition, in superficial and deep gray matter in both presymptomatic and symptomatic neuroferritinopathy. Cavitation of the putamen and globus pallidus increased with disease stage and at follow up. The widespread brain iron deposition in presymptomatic and early disease provides an opportunity for monitoring disease‐modifying intervention.

## Introduction

Neuroferritinopathy (OMIM #606159) is a disorder of neurodegeneration with brain iron accumulation (NBIA type 3), typically presenting in mid‐adulthood with a slowly progressive movement disorder and cognitive impairment.^1^ Most affected individuals have a single base insertion in exon 4 (c460InsA)^2^ of the ferritin light chain (*FTL*) gene but six other mutations in the same gene have been identified.^3^ All variants likely restrict ferritin iron‐sequestering, causing iron deposition and eventual cell death.^4^ The long presymptomatic stage in neuroferritinopathy presents an opportunity for iron chelation before irreversible neurodegeneration.

Brain iron accumulation can be detected in vivo using MRI.^5^, ^6^ Previous studies using 1.5T and 3T MRI in neuroferritinopathy showed iron deposition in the basal ganglia but did not detect the more widespread iron deposition seen even in early disease stages at postmortem.^1^, ^7^, ^8^, ^9^ Other case reports of 3T MRI in neuroferritinopathy show cortical iron deposition, particularly in the motor cortex, and suggest this “pencil sign” may be specific to neuroferritinopathy.^10^, ^11^ Here, we report results using ultra‐high field (7T) MRI in three individuals with a pathogenic *FTL* variant at varying disease stages and a healthy control. Our aim was to determine if 7T MRI could detect brain iron deposition in the superficial and deep gray matter in both presymptomatic and symptomatic neuroferritinopathy.

## Methods

We recruited three participants with the same *FTL* gene mutation (c.460InsA; p.Arg154LysfsTer27): An asymptomatic male in his 30s with a positive family history; a woman in her 50s with a 1‐year history of mild dysarthria, limb ataxia, and dystonia; a man in his 40s with a 10‐year history of orofacial dyskinesia, dysarthria, limb tremor, and dystonia. An asymptomatic woman in her 20s with no family history of neuroferritinopathy or other neurological or psychiatric disease was used as a healthy control. All individuals gave written informed consent to participate (REC 13/YH/0310). The participant with 10 years disease duration returned for a 2‐year follow up assessment and scan. We have not provided detailed demographic information to preserve participant anonymity given the rarity of neuroferritinopathy. None of these patients are included in other published studies.

Participants underwent 7T MRI with a MAGNETOM Terra scanner (Siemens, Germany) including a T_1_‐weighted MP2RAGE structural sequence (*FTL* carriers: 0.7 mm isotropic voxels, TE = 2.64 ms, TR = 3500 ms, TI = 725/3150 ms, Healthy control: 0.75 mm voxels, TE = 1.99 ms, TR = 4300 ms, TI = 840/2370 ms) and multi‐echo T_2_*‐weighted sequence (1.4 mm isotropic resolution, TE_1_ = 4.68 ms, 6 echoes with echo‐spacing 3.24 ms, TR = 43 ms, nominal FA = 15°).

Qualitative review was performed by a neuroradiologist (TM). R_2_* maps were obtained using the Auto‐Regression on Linear Operations algorithm.^12^ QSM maps were derived using the QSMbox 2.0 Multi‐Scale Dipole Inversion algorithm.^13^, ^14^ Tissue segmentation of MP2RAGE images was performed using SPM12 default settings. Cortical regions were derived from the Harvard‐Oxford atlas and subcortical regions from the PD25 atlas.^15^ The dentate region of interest was derived from the Diedrichsen template.^16^ We summarized the R_2_* and QSM concentrations only from voxels predicted as gray or white matter.

We used CSF fraction of the putamen and globus pallidus to measure cavitation in these regions as a postmortem study of neuroferritinopathy confirmed that the cavitation appearance on MRI is caused by loss of brain tissue.^8^ CSF fraction was calculated using SPM12 tissue segmentation maps and the PD25 atlas regions using the spm_summarise function in SPM and Matlab 2021b (Mathworks, USA).

## Results

**Figure 1:**
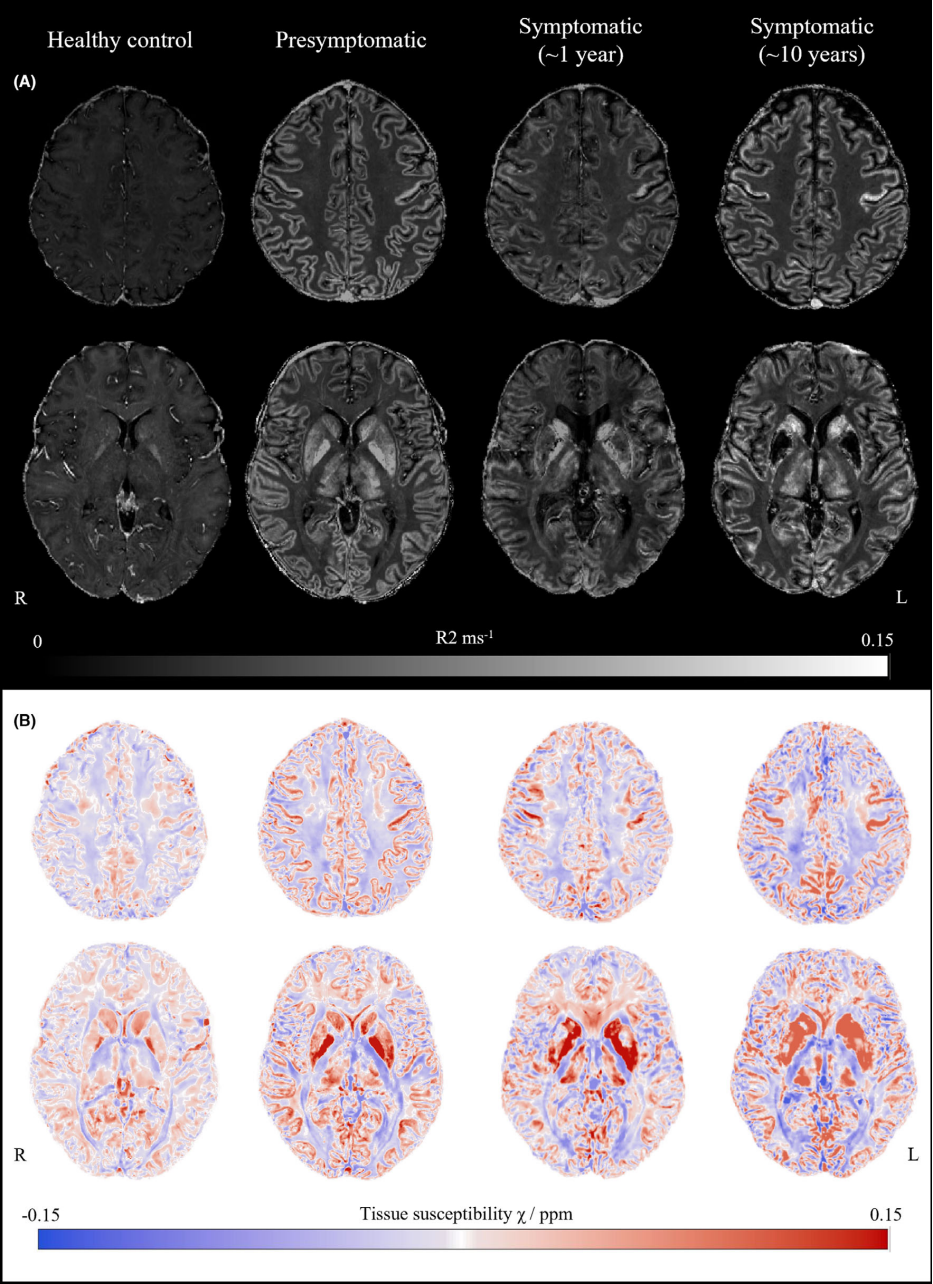
Brain iron accumulation in neuroferritinopathy. (A) Two representative axial R_2_* maps from each participant. Brightness and contrast widths are the same for all images. The R2* images were thresholded at 0.15 ms^−1^ to improve visualization. (B) Two representative axial quantitative susceptibility χ maps from each participant.

In participants with an *FTL* mutation, R_2_* maps demonstrated markedly increased signal compared to controls, consistent with iron deposition, throughout the gray matter of the supratentorial brain. This was most pronounced in the primary motor cortex. There was less involvement of the cerebellum, except in the dentate nucleus where signal was increased in neuroferritinopathy. There was high signal in deep gray matter structures including the putamen, globus pallidus, red nucleus, substantia nigra, and subthalamic nucleus (Fig. 1A). The participants with 10 years of neuroferritinopathy symptoms had higher magnetic susceptibility compared to the presymptomatic *FTL* carrier and individual with 1 year of symptoms. There was no consistent difference in magnetic susceptibility between the presymptomatic *FTL* mutation carrier and the participants with only 1 year of symptoms (Table 1). QSM demonstrated similar intensity distribution to R_2_* maps (Table 1, Fig. 1B). R_2_* and QSM maps were largely unchanged, with minor changes in most regions with formal quantification, in the single neuroferritinopathy participant with a 2‐year interval scan (Table 1).

**Table 1:** Quantification of magnetic susceptibility χ and striatum cavitation (% of CSF in region) in neuroferritinopathy.

	Control	*FTL* mutation carriers			
		Presymptomatic	Symptomatic (1 year)	Symptomatic (10 years)	
				Baseline	2 year follow up
Frontal					
R_2_*	0.019	0.054	0.048	0.064	0.065
QSM	0.018	0.030	0.029	0.037	0.039
Temporal					
R_2_*	0.020	0.058	0.049	0.062	0.062
QSM	0.019	0.031	0.032	0.034	0.034
Parietal					
R_2_*	0.021	0.060	0.048	0.065	0.065
QSM	0.018	0.033	0.030	0.035	0.040
Occipital					
R_2_*	0.022	0.062	0.050	0.066	0.065
QSM	0.020	0.032	0.030	0.036	0.041
Cingulate					
R_2_*	0.020	0.049	0.040	0.058	0.056
QSM	0.020	0.030	0.030	0.044	0.040
Red nucleus					
R_2_*	0.027	0.107	0.077	0.089	0.083
QSM	0.040	0.102	0.208	0.199	0.175
Substantia Nigra					
R_2_*	0.029	0.095	0.066	0.097	0.093
QSM	0.036	0.146	0.161	0.211	0.214
Subthalamic nucleus					
R_2_*	0.027	0.084	0.078	0.074	0.076
QSM	0.019	0.127	0.164	0.094	0.111
Caudate					
R_2_*	0.025	0.062	0.050	0.081	0.084
QSM	0.042	0.057	0.083	0.122	0.110
Putamen					
R_2_*	0.025	0.074	0.064	0.094	0.074
QSM	0.025	0.055	0.081	0.133	0.113
%CSF	0.039	0.124	0.087	0.096	0.078
Globus pallidus					
R_2_*	0.094	0.208	0.199	0.209	0.167
QSM	0.022	0.073	0.055	0.080	0.080
%CSF	0.021	0.039	0.049	0.047	0.051
Thalamus					
R_2_*	0.019	0.054	0.048	0.064	0.065
QSM	0.018	0.030	0.029	0.037	0.039
Dentate nucleus					
R_2_*	0.023	0.058	0.054	0.079	0.079
QSM	0.021	0.038	0.075	0.101	0.108

Susceptibility values (QSM) in ppm. R_2_* values in ms‐1. %CSF values are percentage of region containing CSF (a proxy for cavitation).

**Figure 2:**
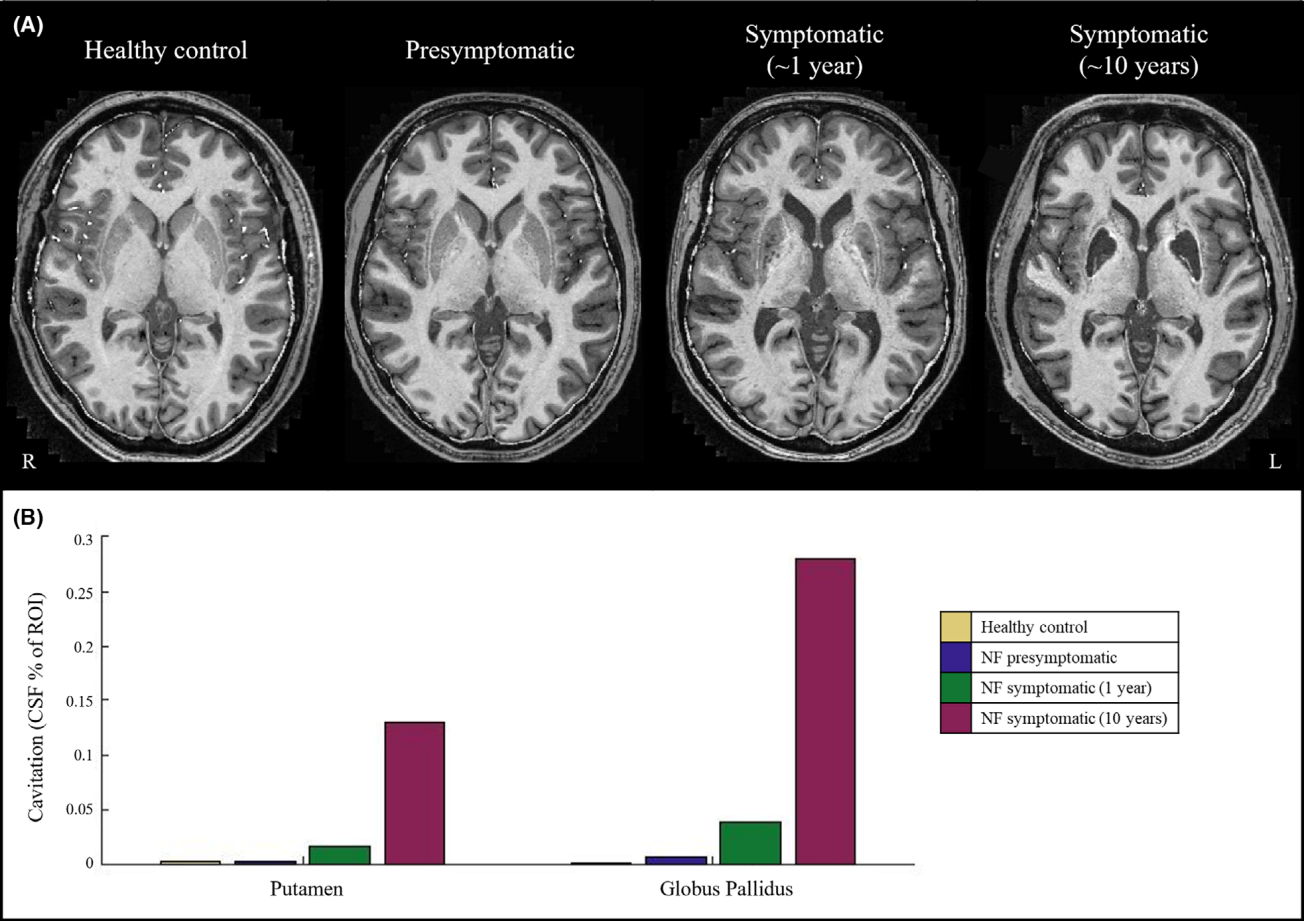
Cavitation in neuroferritinopathy. (A) Representative axial imaging from T_1_‐weighted MP2RAGE images in MNI space. (B) Cavitation (measured by CSF fraction of the region of interest) in the putamen and globus pallidus.

CSF replacement of brain tissue (cavitation) in the putamen and globus pallidus increased with disease duration in neuroferritinopathy (Fig. 2). There was no cavitation in the healthy control nor the presymptomatic *FTL* mutation carrier. Early cavitation was apparent in the patient with 1 year of symptoms and severe cavitation after 10 years disease duration which then increased over 2 years (Table 1).

## Discussion

Ultra‐high field MRI shows R_2_* and QSM values are increased, suggesting iron accumulation, throughout the brain in neuroferritinopathy. This was already widespread in the presymptomatic participant. More advanced disease was associated with greater cavitation of putamen and globus pallidus but there was not a clear increase in iron deposition.

We show that 7T MRI can detect the iron deposition in deep and superficial gray matter, seen at postmortem but not with lower field (1.5T) MRI.^1^, ^3^, ^8^, ^9^, ^10^ 7T MRI revealed involvement of widespread cortical regions, including the motor cortex, and deep gray matter structures, including the red nucleus and substantia nigra.

There was no increase in iron deposition between participants with different disease stages, or in the single case with longitudinal follow up. In contrast, there was a clear association between cell loss in the striatum and more advanced disease. The presymptomatic participant had widespread iron but no striatal cavitation while symptomatic participants had a similar extent of iron deposition but severe cavitation, which increased with follow up. This suggests that it is the cell loss which eventually follows iron deposition that causes the clinical features of neuroferritinopathy. This is supported by previous findings of iron deposition in early childhood, decades before anticipated symptom onset^7^ and postmortem evidence of a presymptomatic case with widespread brain iron deposition but no neuronal loss.^8^ Alternatively, any increase in iron deposition may be undetected by MRI due to a ceiling effect of magnetic susceptibility. This has been suggested in other NBIAs with postmortem validation^17^ but not in neuroferritinopathy.^8^

The widespread iron deposition and subsequent neurodegeneration mirrors the clinical presentations of neuroferritinopathy and explains why many symptoms are not typically associated with a focal disease of the basal ganglia.^1^ Limb stiffness has been attributed to dystonia but can be associated with pyramidal signs^1^ which would be explained by iron accumulation in the motor cortices. The extensive cortical iron deposition explains the heterogenous cognitive impairments which include executive dysfunction, disinhibition, emotional lability, psychosis, and apraxia.^18^

Our study has limitations. This was a pilot study with only 3 *FTL* mutations carriers and a single control with unmatched demographics. Only a single participant had longitudinal imaging. This prevents any definitive conclusions on the relationship between iron accumulation, confounders (e.g., age and sex), and clinical phenotype. The control participant was younger than any of the *FTL* mutation carriers which may confound our results as brain iron accumulates with age. We used magnetic susceptibility as a measure of brain iron but this can be affected by other substances including myelin. Our results are corrected for brain atrophy, but a larger study would enable more robust statistical analysis of the relationship between brain atrophy and magnetic susceptibility.

In conclusion, this pilot study using ultra‐high field MRI suggests neuroferritinopathy is associated with widespread superficial and deep gray matter iron deposition before symptom onset. Neuroferritinopathy has a presymptomatic stage of decades,^7^ which we show can be detected with ultra‐high field MRI and provides an opportunity for disease modification before irreversible neurodegeneration and striatal cavitation. Iron chelation is a promising treatment for neuroferritinopathy, with a causative link between *FTL* mutation, iron accumulation, and neurodegeneration.^3^, ^4^ Our pilot study supports the use of ultra‐high field MRI as a trial endpoint,^1^, ^9^ similar to other NBIA subtypes.^19^ Larger studies are required to test the association between regional variation in iron deposition and clinical phenotype.

## Funding Information

This work was funded by the UK Medical Research Council (13044). A.G. Murley is an NIHR Clinical Lecturer. C. T. Rodgers was funded by a Sir Henry Dale Fellowship from the Wellcome Trust and Royal Society (098436/Z/12/B). J. van den Ameele is a Wellcome Clinical Research Career Development Fellow (219615/Z/19/Z), who receives support from the Evelyn Trust (21–25), the MRC Mitochondrial Biology Unit (MC_UU_00028/8), and the BBSRC (BB/X00256X/1). R.Horvath. is a Wellcome Trust Investigator (109915/Z/15/Z), who receives support from the Medical Research Council (UK) (MR/V009346/1), the Addenbrookes Charitable Trust (G100142), the Evelyn Trust, the Stoneygate Trust, the Lily Foundation, Action for AT and an MRC strategic award to establish an International Centre for Genomic Medicine in Neuromuscular Diseases (ICGNMD) MR/S005021/1. P.F. Chinnery is a Wellcome Trust Principal Research Fellow (212219/Z/18/Z), and a UK NIHR Senior Investigator, who receives support from the Medical Research Council Mitochondrial Biology Unit (MC_UU_00028/7), the Medical Research Council (MRC) International Centre for Genomic Medicine in Neuromuscular Disease (MR/S005021/1), the Leverhulme Trust (RPG‐2018‐408), an MRC research grant (MR/S035699/1), and an Alzheimer's Society Project Grant (AS‐PG‐18b‐022). This research was supported by the NIHR Cambridge Biomedical Research Centre (NIHR203312). The views expressed are those of the author(s) and not necessarily those of the NIHR or the Department of Health and Social Care.

## Author Contributions

AGM substantially contributed to analysis and interpretation of data and drafted the manuscript. CR, HB, JvdA, and RH substantially contributed to acquisition, analysis and interpretation of data and reviewed the manuscript for important intellectual content. CTR and TM substantially contributed to analysis and interpretation of data and reviewed the manuscript for important intellectual content. PFC conceived and designed the study, substantially contributed to analysis and interpretation of data and drafted the manuscript.

## Conflict of Interest

Catarina Rua is an employee of Invicro, London, UK. None of the other authors have any conflicts of interest relevant to this study.

## Data Availability

Anonymized data are available on reasonable request to the lead author for academic (noncommercial) purposes, although restrictions may apply to adhere to participant consent and anonymity.

## References

[1] Chinnery PF , Crompton DE , Birchall D , et al. Clinical features and natural history of neuroferritinopathy caused by the FTL1 460InsA mutation. Brain. 2007;130(1):110‐119. 17142829 10.1093/brain/awl319

[2] Curtis ARJ , Fey C , Morris CM , et al. Mutation in the gene encoding ferritin light polypeptide causes dominant adult‐onset basal ganglia disease. Nat Genet. 2001;28(4):350‐354. 11438811 10.1038/ng571

[3] Keogh MJ , Morris CM , Chinnery PF . Neuroferritinopathy. International Review of Neurobiology. Academic Press Inc.; 2013:91‐123. 10.1016/B978-0-12-410502-7.00006-5 24209436

[4] Levi S , Rovida E . Neuroferritinopathy: from ferritin structure modification to pathogenetic mechanism. Neurobiol Dis. 2015;81:134‐143. 25772441 10.1016/j.nbd.2015.02.007 PMC4642653

[5] Lehéricy S , Roze E , Goizet C , Mochel F . MRI of neurodegeneration with brain iron accumulation. Curr Opin Neurol. 2020;33(4):462‐473. 32657887 10.1097/WCO.0000000000000844

[6] Tambasco N , Nigro P , Chiappiniello A , et al. An updated overview of the magnetic resonance imaging of brain iron in movement disorders. Behav Neurol. 2022;2022:1‐20. 10.1155/2022/3972173 PMC8894064 35251368

[7] Keogh MJ , Jonas P , Coulthard A , Chinnery PF , Burn J . Neuroferritinopathy: a new inborn error of iron metabolism. Neurogenetics. 2012;13(1):93‐96. 22278127 10.1007/s10048-011-0310-9 PMC4038507

[8] Kurzawa‐Akanbi M , Keogh M , Tsefou E , et al. Neuropathological and biochemical investigation of hereditary Ferritinopathy cases with ferritin light chain mutation: prominent protein aggregation in the absence of major mitochondrial or oxidative stress. Neuropathol Appl Neurobiol. 2021;47(1):26‐42. 32464705 10.1111/nan.12634

[9] McNeill A , Gorman G , Khan A , Horvath R , Blamire AM , Chinnery PF . Progressive brain iron accumulation in neuroferritinopathy measured by the thalamic T2* relaxation rate. Am J Neuroradiol. 2012;33(9):1810‐1813. 22499840 10.3174/ajnr.A3036 PMC4038493

[10] Batla A , Adams ME , Erro R , et al. Cortical pencil lining in neuroferritinopathy: a diagnostic clue. Neurology. 2015;84(17):1816‐1818. doi:10.1212/WNL.0000000000001511 25832658 PMC4424124

[11] Lehn A , Mellick G , Boyle R . Teaching NeuroImages: Neuroferritinopathy. 2011 https://www.neurology.org 10.1212/WNL.0b013e318236492c 22042801

[12] Pei M , Nguyen TD , Thimmappa ND , et al. Algorithm for fast monoexponential fitting based on auto‐regression on linear operations (ARLO) of data. Magn Reson Med. 2015;73(2):843‐850. 24664497 10.1002/mrm.25137 PMC4175304

[13] Acosta‐Cabronero J , Milovic C , Mattern H , Tejos C , Speck O , Callaghan MF . A robust multi‐scale approach to quantitative susceptibility mapping. NeuroImage. 2018;183:7‐24. 30075277 10.1016/j.neuroimage.2018.07.065 PMC6215336

[14] Rua C , Clarke WT , Driver ID , et al. Multi‐centre, multi‐vendor reproducibility of 7T QSM and R2* in the human brain: results from the UK7T study. NeuroImage. 2020;223:117358. 32916289 10.1016/j.neuroimage.2020.117358 PMC7480266

[15] Xiao Y , Fonov V , Bériault S , et al. Multi‐contrast unbiased MRI atlas of a Parkinson's disease population. Int J Comput Assist Radiol Surg. 2015;10(3):329‐341. 24841147 10.1007/s11548-014-1068-y

[16] Diedrichsen J , Balsters JH , Flavell J , Cussans E , Ramnani N . A probabilistic MR atlas of the human cerebellum. NeuroImage. 2009;46(1):39‐46. 19457380 10.1016/j.neuroimage.2009.01.045

[17] Vroegindeweij LHP , Wielopolski PA , Boon AJW , et al. MR imaging for the quantitative assessment of brain iron in aceruloplasminemia: a postmortem validation study. NeuroImage. 2021;245:118752. 34823024 10.1016/j.neuroimage.2021.118752

[18] Keogh MJ , Singh B , Chinnery PF . Early neuropsychiatry features in neuroferritinopathy. Mov Disord. 2013;28(9):1310‐1313. 23436236 10.1002/mds.25371

[19] Klopstock T , Tricta F , Neumayr L , et al. Safety and efficacy of deferiprone for pantothenate kinase‐associated neurodegeneration: a randomised, double‐blind, controlled trial and an open‐label extension study. Lancet Neurol. 2019;18(7):631‐642. https://linkinghub.elsevier.com/retrieve/pii/S1474442219301425 31202468 10.1016/S1474-4422(19)30142-5

